# Depression and its associated factors among primary caregivers of adult cancer patients at Northwest Amhara Regional States Referrals Hospitals oncology treatment units, Northwest Ethiopia, 2021

**DOI:** 10.1186/s12888-022-04182-w

**Published:** 2022-08-06

**Authors:** Likinaw Abebaw Wassie, Abere Woretaw Azagew, Berhanu Boru Bifftu

**Affiliations:** 1 Department of Medical Nursing, School of Nursing, College of Medicine and Health Sciences, University of Gondar, Gondar, Ethiopia; 2 Community Health Nursing Unit, School of Nursing, College of Medicine and Health Sciences, University of Gondar, Gondar, Ethiopia

**Keywords:** Depression, Prevalence, Primary caregivers, Bahir Dar, Gondar, Ethiopia

## Abstract

**Introduction:**

Cancer is a primary and challenging health problem, has a significant impact on caregivers, and is a leading cause of emotional responses like depression. Depression is the most common and serious psychiatric disorder that has a considerable effect on the daily life of primary caregivers. Different articles reported that the magnitude of depression was prevalent among primary caregivers of patients with cancer. There is scarcity of published articles about the problem in Ethiopia. This study aimed to assess the prevalence of depression and associated factors among primary caregivers of adult cancer patients at Northwest Amhara Regional States Referrals Hospitals, oncology treatment units, Northwest Ethiopia, 2021.

**Methods:**

An institutional-based cross-sectional study was conducted in Northwest Amhara Regional States Referrals Hospitals. A systematic random sampling technique was used to select 421 participants. The data were collected using interviewer-administered and chart review through structured, pretested Patient Health Questionarie-9 questionnaires. The data were entered into Epi. Data version 4.6 and analyzed using Stata version 14.0. Bivariable and multivariable logistic regression were carried out to identify factors associated with depression. Adjusted odds ratio with a 95% confidence interval and variables with a *p*-value < 0.05 were considered significantly associated with depression.

**Results:**

The prevalence of depression was 45.15%. Being female (AOR = 2.01, 95%, CI: 1.27–3.19), having an average monthly income of < 1000 Ethiopian birr (AOR = 3.54, 95% CI: 1.54–8.13), and primary caregivers of a cancer patient with mixed therapy (AOR = 4.54, 95%, CI: (1.5–13.48) were significantly associated with depression.

**Conclusion:**

The prevalence of depression among primary caregivers of adult patients with cancer was significant. It’s better, health care providers in oncology treatment facilities need to recognize and screen and give special attention to female primary caregivers, having an average monthly income of < 1000 Ethiopian birr, and primary caregivers of a cancer patient with mixed therapy to reduce the risk of developing depression.

## Background

All over the world, cancer is a dominant public health problem and a cardinal cause of death worldwide, accounting for one-sixth of all deaths. It has been estimated more than 9.6 million attributable deaths worldwide in 2018 [1]. The developing countries of the world also accounted for 65% of cancer deaths. Sub-Saharan Africa (SSA) countries are commonly featured on the list of developing countries of the world, and nowadays they are experiencing a major cancer burden [1].

Globally there were an estimated 18.1 million existing new cases of cancer every year [2]. About 70% of all cancer deaths have existed in low- and middle-income countries, including Ethiopia [3, 4]. According to Ethiopian Population-Based Registry Data in 2015, estimated that 21,563 incident cancer cases were diagnosed in males and females, respectively [5]. In Ethiopia there is only one cancer center; the rest of the others are oncology treatment units rather than oncology centers. The treatment outcome of cancer patients depends on the stage and type of cancer, the biology of the tumor, and patient factors. Accordingly, the ultimate goal of cancer treatment is to relieve the pain of the patients [6].

The diagnosis of cancer is a terrible and the most stressful event that has a huge influence on patients and their primary caregivers and may cause emotional responses of hopelessness, helplessness, worthlessness, guilty feeling, irritability, and depression [7]. It has a serious brunt on the physical, emotional, and practical aspects of the life of patients and their primary caregivers. As cancer treatment alters to a more entanglement and advanced stage, the patients’ multitudinous needs have blown up from treatment monitoring and symptom management to emotional, psychological, and financial aid and assistance with personal care [8]. During the disease and treatment, the family is the most involved and necessary group in caring for the patient; helping them adapt and manage their disease [9].

Around 3 million people are provided as primary caregivers to patients with cancer in the United States by the year [10]. Cancer care is progressively delivered on an outpatient basis with a higher number of caregiving burdens falling to informal caregivers. “Cancer primary caregivers put in an average of 32.9 h per week on caregiving roles and 72% perform multiple medical or nursing tasks” [11]. Approximately two-thirds (62%) of caregivers are in a “high burden’’ condition and the average burden of care is higher for cancer than for non-cancer primary caregivers [12].

The effect of these illnesses on the primary caregivers of patients with cancer is substantial. Caregiver depression is a mood disturbance resulting from the stress of providing care, which may be manifested as persistent sadness and a loss of interest in activities that one normally enjoys, accompanied by an inability to conduct daily activities, for a minimum of two weeks. It is an important and common adverse repercussion, a poor quality of life (QOL), and is a troubling factor for other disorders [13].

Different studies revealed that most primary caregivers of patients with cancer are felt depressed and primary caregiver burden [14, 15]. A recent study showed that more than 60% of primary caregivers are affected by depression, sadness, and frustration while providing care [16]. Approximately 50% of the people with depression do not receive any management for their depression in developing countries, thus increasing the depressive magnitude in those countries [17]. Previous literature has reported that the prevalence rate for primary caregiver depression ranged from 20 to 73% and the higher level of depression in primary caregivers is frequently associated [18], with socio-demographic characteristics, clinical characteristics of the patient, primary caregiver burden, and behavioral factors of primary caregivers [17, 19–22].

A previous study conducted in Ethiopia among primary caregivers of adult cancer patients reported that the prevalence of depression was 54% [23]. Different methods are applied to decrease the prevalence of depression among primary caregiver such as a community-based psychoeducationa, providing emotional support, problem solving and skills building, supportive therapy, family or couples therapy, cognitive-behavioral therapy, complementary and alternative medicine are implemented [24].

Depression distorts the quality of life of primary caregivers in different ways, but the greatest burden is related to their psychological well-being. Furthermore, psychological services are not available for primary caregivers to access at the cancer centers. However, there are little data known about depression among primary caregivers of patients with cancer in African countries including Ethiopia. Therefore, this study was to determine the magnitude of depression among primary caregivers of patients with cancer and its associated factors.

## Methods

### Study design and period

An institutional-based cross-sectional study was implemented from March 15 to May 15, 2021.

### Study setting

The study was conducted in Northwest Amhara Regional States Referrals Hospitals. There are a total of five Referral Hospitals in Northwest Amhara Referral Hospitals; Debre Markos, Felege Hiwot, Tibebe Gion, Debre Tabor, and the University of Gondar found in the Northwest of Amhara. Each Referral Hospital serves 3.5–5 million people [25]. Of the five Referral Hospitals, the two (University of Gondar Comprehensive Specialized Hospital (GUCSH) and Felege Hiwot Comprehensive Specialized Hospital (FHCSH)) have oncology treatment units. Those two Referral Hospitals are located in the Amhara Regional State, Northwest Ethiopia 738 km and 565 km away from the capital city of Ethiopia: Addis Ababa respectively. There are a total of 880 cancer patients in the two Referral Hospitals. The oncology treatment unit of GUCSH was established in 2014 G.C and currently has 450 cancer patients and 17 beds for the management of cancer patients. Whereas the oncology treatment unit of FHRH was established in 2016 G.C and has 430 cancer patients; currently has 18 beds for inpatient treatment of cancer patients. A one-month average number of cancer patients who had follow-up treatments in GUCSH and FHRH were 230 and 210 respectively.

### Study participants

All primary caregivers of adult cancer patients who were ≥ 18 years old and who had been providing care for at least two weeks were included in the study. Those Primary caregivers who had a history of known depression disorder before being a caregiver and who are unable to hear or speak were excluded from the study.

### Sample size determination and sampling procedure

The sample size was calculated using the single population proportion formula; considering the following: 95% confidence interval (CI), 54% proportion of depression from the previous study [23], and 5% margin of error. The final sample size was 421 considering a 10% non-respondent rate. The sample size was proportionally allocated for each selected referral hospitals based on their number of cancer patients. Prior to the data collection period the number of primary caregivers were estimated based on the report obtained from the oncology treatment units of each selected hospital at least one primary caregiver comes with the cancer patients in every schedule. Considering this, on average the total number of primary caregivers is equivalent to the number of cancer cases. Then, the k value was calculated dividing by total care givers to sample size (K = N/n, where; K = the interval, *N* = total number of primary caregivers). The starting unit was determined by lottery method which was 2 and then, using systematic random sampling the study participant was selected every 2 person pattern.


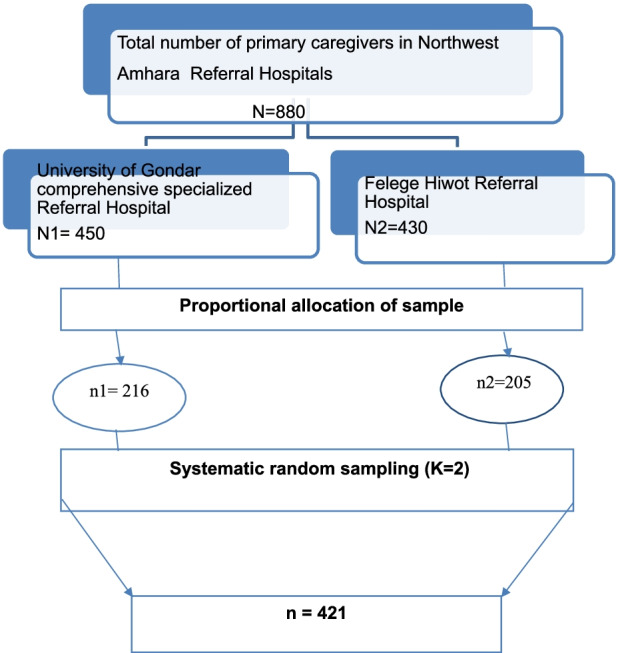


### Operational definition

Depression in caregivers- depression in caregivers were measured by using the PHQ-9 depression assessment tool. Those caregivers who score greater than or equal to 10 were taken as depressed [26, 27].

Primary caregivers- are family members and/or close friends who care for the patient. Family members can be father, mother, sister, brother, daughter, son, uncle, ant, grandfather or mother, partner, son or daughter in law and other blood relatives who were not be paid for caring the patient and should be the one who stays with patient caring for two weeks and above [28–30].

### Data collection instruments and procedures

The data were collected using interviewer-administered and chart review through structured pretested questionnaires that are adapted from a questionnaire developed by a previous study, which contains five sections the first section contains ten questions regarding socio-demographic characteristics of the study participants, the second section contains nine questions related to clinical characteristics of a patient. the third section contains three questions regarding primary caregivers related, the fourth section contains eight questions related to primary caregiver's behavior, and the fifth section contains nine questions(PHQ-9) for the assessment of depression [31]. PHQ-9 has been validated for screening and diagnosis of depression in patients [32], primary caregivers [33], and population-level [34]; it is also validated in Ethiopia's health care context with specificity and sensitivity of 67% and 86% respectively [20, 33]. The data were collected by four BSc nurses who have working experience in the oncology treatment unit. Primary caregivers were identified by asking the patient who is their primary caregiver and provides consistent care and then if the primary caregiver who fulfilled the inclusion criteria were interviewed after guarantying their willingness to take part in the study.

### Data processing and analysis

After data collection, the collected data were cleaned and checked for completeness. Data were entered by using Epi data version 4.6, after being coded and analyzed using Stata version 14.0. Descriptive statistics were used in the analysis of medians, frequencies, and percentages were computed for all variables. Data were presented in tables and graphs. The association between dependent and independent variables was assessed by using a binary logistic regression analysis model, to estimate the strength of association using Odds Ratios (OR). All variables associated with depression with a *p*-value less than 0.2 in the bivariable analysis, were further analyzed using multivariable analyses to control potential confounding factors. Variables with a *p*-value less than 0.05 were declared to be associated with depression.

### Data quality control

A pre-test was done on 5% of the total sample size to make sure whether the questionnaire is appropriate and to ensure its validity in the study population before the actual data collection time. The reliability test was performed for PHQ-9 questionnaires using Cronbach’s alpha and initially, the value was 0.879. After the pretest training were given to all data collectors and supervisors on the purpose of the study, how to get informed consent, and the technique of selecting the study participants from each oncology treatment unit. Supervision were conducted by the supervisors and Principal Investigator. All questionnaires were translated into local languages (Amharic) before data collection. Consistency was checked by a back-translation by another expert fluent both in English and in local languages. At the end of each data collection day, the supervisors were checked for completeness or fulfillment of the questionnaires and the quality of the recorded information.

### Ethical consideration

Ethical clearance was obtained from the School of Nursing research ethical review commute on the behalf of the University of Gondar institutional review board. Written permission letters were obtained from Hospital managers. Participants were informed about the purpose of the study and written informed consent was obtained from them. Confidentiality was maintained by omitting direct personal identifiers on the questionnaire, using code numbers, storing data locked with a password, and not misusing or disclosing their information. Participants were also informed that participation was voluntary and that they have the right to withdraw from the study participation at any stage if they are not comfortable with the investigation. The issue of privacy and confidentiality was strictly maintained. All methods were carried out in accordance with relevant guidelines and regulations.

## Results

### Socio-demographic characteristics of the respondents

A total of 421 study participants were enrolled in the study with a response rate of 412 (97.86%). The Median age of the study participant was 35 with an Inter-Quartile Range (IQR) of 28–46.5 years. Nearly one-third 141 (34.22%) of them were between the age of 29–39 years. More than half (57.28%) of the study participants were male and the majority 327 (79.37%) of the respondents were followers of orthodox Christianity. Most 395 (95.87%) of the study participants were Amhara by ethnicity and two hundred thirty-two (56.31%) were urban dwellers. (Table 1).

**Table 1:** Socio-demographic characteristics of primary caregivers of adult cancer patients in Northwest Amhara Regional States Referrals Hospitals, oncology treatment units, July/ 2021. (*n* = 412)

Variables	Category	Frequency(n)	Percent (%)
Age	18–28	110	26.70
	29–39	141	34.22
	40–50	98	23.79
	51–60	51	12.38
	> = 61	12	2.91
Sex	Male	236	57.28
	Female	176	42.72
Religion	Orthodox	327	79.37
	Muslim	80	19.42
	Protestant/Catholic	5	1.21
Ethnicity	Amhara	395	95.87
	Kimant	17	4.13
Marital status	Single	109	26.46
	Married	291	70.63
	Divorced/Widowed	12	2.91
Educational status	Unable to read and write	102	24.76
	Primary(1–8)	73	17.72
	Secondary (9–12)	133	32.28
	Collage and above	104	25.24
Occupation	Governmental employed	74	17.96
	NGO employed	26	6.31
	Farmer	129	31.31
	Merchant	53	12.86
	Student	63	15.29
	Housewife	47	11.41
	Unemployed/daily labor	20	4.85
Residence	Urban	232	56.31
	Rural	180	43.69
Relationship	Spouse	140	33.98
	Child	150	36.41
	Siblings	85	20.63
	Parents	21	5.1
	Other relatives	16	3.88
Average monthly income(in ETB)	< = 1000	90	21.84
	1000–1999	78	18.93
	2000–2999	55	13.35
	> 3000	189	45.87

*Note* _ *NGO* Non-Governmental Organization, *ETB* Ethiopian Birr

### Clinical-related characteristics of patients

Among a total of 412 cancer cases, around 31 types of cancer are listed; breast cancer 91(22.09%), cervical cancer 54(13.11%), gastric cancer 30(7.28%), and else. More than one-third of 165 (40.05%) of the patient had stage four (IV) cancer. Most 410 (99.51%) of patients were taking cancer treatment and the majority 351(85.19%) of patients were taking chemotherapy. Nearly one-thirds 115 (27.91%) of the patient had known comorbid illness. More than one-third 38(33.04%) of the patient had hypertension. (Table 2).

**Table 2:** Clinical-related characteristics of adult cancer patients in Northwest Amhara Regional States Referrals Hospitals, oncology treatment units, July/2021. (*n* = 412)

Variables	Category	Frequency(n)	Percent (%)
Clinical stage	Stage I	19	4.61
	Stage II	90	21.84
	Stage III	138	33.5
	Stage IV	165	40.05
Duration since diagnosis	< 6 Months	139	33.74
	7–12 Months	123	29.85
	> 12 Months	150	36.41
Does the patient take treatment?	Yes	410	99.51
	No	2	0.49
Type of treatment	Chemotherapy	344	83.50
	Hormonal therapy	33	8.01
	Mixed	24	5.83
	Surgery only	11	2.67
Duration since the start of treatment	1–3 Months	77	18.69
	4–6 Months	77	18.69
	> 6Months	258	62.62
Comorbidity	Yes	115	27.91
	No	297	72.09
Type of comorbidity	Anemia	30	26.09
	HIV/AIDS	21	18.26
	Hypertension	38	33.04
	Diabetes	19	16.52
	CHF	8	6.96
	Asthma	6	5.22
ECOGP	Grade 0	28	6.83
	Grade 1	77	18.78
	Grade 2	171	41.71
	Grade 3	126	30.73
	Grade 4	8	1.95
Are you living with your relatives	Yes	319	77.43
	No	93	22.57
Do you know the diagnosis of the disease	Yes	404	98.06
	No	8	1.94
Time off caring per day/Hours	< = 6 Hours	165	40.05
	> 6 Hours	247	59.95

*Note*_ *ECOGP* Eastern Cooperative Oncology Group Performance, *CHF* Congestive Heart Failure

### Behavioral related characteristics of participants

**Fig. 1:**
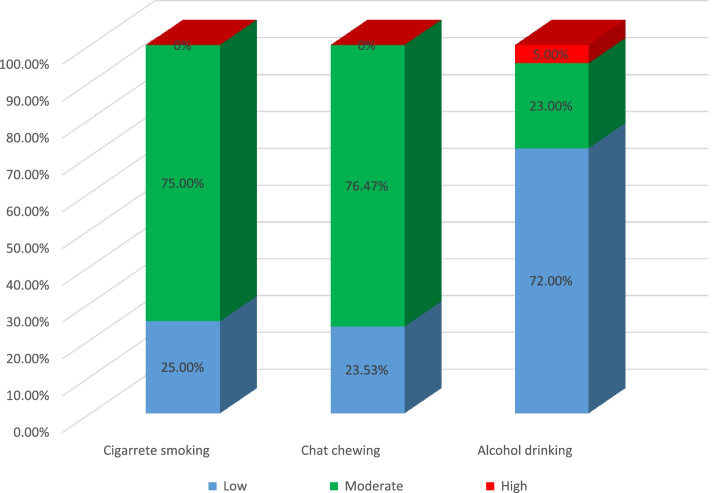
Behavioral-related characteristics of primary caregivers of adult cancer patients in Northwest Amhara Referral Hospitals, oncology treatment units, 2021

Of the total respondents (*n* = 412); one hundred (24.27%) and seventeen (4.13%) of the participants had a history of alcohol drinking and chat chewing respectively. Only eight (1.94%) of the respondent had ever used cigarette smoking. Nearly three fourth (72%) of participants who had a history of alcohol use were found to have a low score of specific substance involvement. Three fourth (75%) and a bit more than three fourth (76.47%) of participants who had a history of cigarette smoking and chat chewing were found to have a moderate score of specific substance involvement respectively. (Fig. 1).

### The overall prevalence of depression

**Fig. 2:**
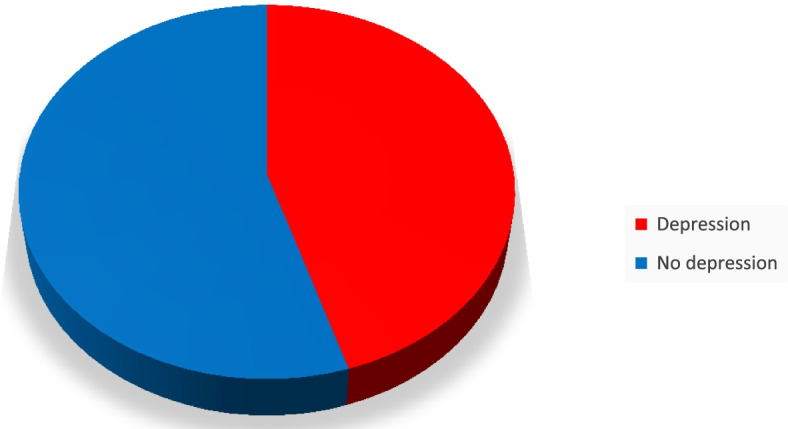
The overall prevalence of depression among primary caregivers of adult cancer patients, July /2021. (*n* = 412)

The prevalence of depression among primary caregivers of adult cancer patients in Northwest Amhara Referral Hospitals and oncology treatment units was found to be 45.15% with (95% CI: 40.38–50.001). Of which accounted for females 22.82%.( Fig. 2).

### Factors associated with depression

Bivariate analysis was carried out to identify factors associated with depression among primary caregivers of adult cancer patients. Sex, marital status, education, occupation, residency, relationship, comorbidity, type of treatment, alcohol use throughout life, and average monthly income were significantly associated with depression. Finally, multivariable analyses were conducted and sex, type of treatment, and having an average monthly income < 1000 ETB were significantly associated with depression among primary caregivers of adult cancer patients. Female primary caregivers were nearly two times more likely to develop depression as compared to males ( AOR = 2.01, 95%, CI: 1.27–3.19), having average monthly income < 1000 ETB were nearly four times more likely to develop depression as compared to their counterparts (AOR = 3.54, 95% CI: 1.54–8.13), primary caregivers of a cancer patient with mixed therapy were nearly five times more likely to develop depression as compared to primary caregivers whose cancer patient with chemotherapy only (AOR = 4.54, 95%, CI: (1.5–13.48). (Table 3).

**Table 3:** Factors associated with depression among primary caregivers of adult cancer patients, July/2021. (*n* = 412)

Variables	Category	Depression	COR (95% CI)	AOR(95%CI)	*P* -value
		No Yes			
Sex	Male Female	144 92 82 94	1.00 2.79(1.21–2.66)	1.00 **2.01(1.27–3.19)**	0.003*
Marital status	Single Married Divorced/widowed	68 41 151 140 7 5	1.00 1.54(0.98–2.41) 1.18(0.35–3.98)	1.00 1.40(0.71–2.77) 0.85(0.21–3.41)	0.330 0.817
Education	Unable to read and write Primary(1–8) Secondary (9–12) Collage and above	52 50 36 37 74 59 64 40	1.00 1.07(0.59–1.95) 0.83(0.49–1.39) 0.65(0.37–1.13)	1.00 1.54(0.76–3.06) 1.63(0.70–3.78) 1.38(0.43 -4.38)	0.218 0.255 0.584
Occupation	Governmental employed NGO employed Farmer Merchant Student Housewife Others	40 34 17 9 69 60 34 19 35 28 19 28 12 8	0.98(0.55–1.73) 0.61(0.25–1.47) 1 0.64(0.33–1.24) 0.92(0.50–1.69) 1.69(0.86–3.33) 0.77(0.29–2.00)	2.55(0.80–8.10) 1.96(0.46–8.35) 1 1.45(0.49–4.25) 1.77(0.61–5.10) 1.45(0.59–3.53) 2.26(0.60–8.54)	0.118 0.360 0.499 0.290 0.416 0.231
Residency	Urban Rural	137 95 89 91	1 1.47(1.00–2.18)	1 1.38(0.54–3.48)	0.499
Relationship	Spouse Child Siblings Parents/other relative	69 71 87 63 53 32 17 20	1 0.70(0.44–1.12) 0.59(0.34–1.02) 1.14(0.55–2.36)	1 0.70(0.38–1.28) 0.59(0.31–1.11) 1.04(0.47–2.27)	0.244 0.102 0.926
Comorbidity	Yes No	56 59 170 127	1.41(0.92–2.17) 1	1.50(0.91–2.49) 1	0.113
Type of treatment	Chemotherapy Hormonal therapy Mixed Surgery	193 151 22 11 5 19 6 5	1 0.64(0.30–1.36) 4.86(1.77–13.31) 1.07(0.32–3.56)	1 0.67(0.29–1.52) **4.54(1.5–13.48)** 1.30(0.35–4.76)	0.336 0.006* 0.693
Alcohol use	Yes No	48 52 178 134	1.44(0.92–2.26) 1	1.21(0.71–2.06) 1	0.482
Average monthly income	< 1000 1000–1999 2000–2999 > 3000	37 53 43 35 29 26 117 72	2.32(1.39–3.89) 1.32(0.78–2.26) 1.46(0.80–2.67) 1	**3.54(1.54–8.13)** 1.58(0.75–3.32) 1.46(0.73–2.95) 1	0.003^*^ 0.231 0.286

*P* = 0.05 statically significant

## Discussion

This study determined the prevalence of depression and its associated factors among primary caregivers of adult cancer patients in Northwest Amhara Regional States Referrals Hospitals oncology treatments units. The overall prevalence of depression among study participants was found to be 45.15% with (95% CI:40.38–50.001). The prevalence of depression found in this study was slightly lower than the findings of a study conducted in Ethiopia: Hawassa 54% [23]. The possible reason for this difference may be due to the difference in sampling method, such as in this study systematic random sampling was used, while a study conducted in Hawassa used a convenient sampling method. Similarly, the result was also lower than the studies done among primary caregivers of patients with cancer revealed in Kenya at 62.7% [35], China at 63.5% [22], and Korea at 67% [36], and another study was conducted in Korea 57.6%, 51.2% [37]. The inconsistency between our findings and that of these studies might be due to Methodological differences, especially sensitivity differences in the different screening tools. While in this study PHQ-9 was used, studies conducted in china used CESDS, Korean and Kenya study was used BDI.

On the contrary, the prevalence of depression in this study was higher than studies conducted in Uganda 26% [17], Malaysia 29.4% [38], Turkey 33.3%% [39], another study conducted in Turkey 29% [40], Iran 31.2% [8], Midwestern United State 37.5% [41] and another study conducted in United State 26.9% [42]. The difference might be due to the difference in study design. In this study cross-sectional study design was used, while a study conducted in Turkey, Midwestern USA and US a prospective cohort study design was used. This variation could also be due to differences in the socio-economic, cultural, and lifestyle differences between those countries. The possible reason may be due to health care delivery policy differences and the country’s priority to cancer treatment and prevention in the case of the Midwestern United States and Turkey [43]. Moreover, a large proportion of patients with cancer in this study area come to seek health care in the late stage cancer-causing depression to their primary caregivers.

Regarding the associated factors of depression among primary caregivers of adult patients with cancer, female primary caregivers were nearly two times more likely to develop depression as compared to males. This finding was supported by studies conducted in the Midwestern United state [21], Asia [9], Korea [36], and china [4]. This might be due to that the majority of societies perceive and trusts the female caregivers to be more caring than their male counterparts and also household management roles and tasks or indoor activities are engaging for females [44]. Male primary caregivers face their care-related challenges in a robust way, such as decreasing disruptions, targeted on tasks, and retaining their stress to themselves. The possible justification might be due to: females by nature are nurturers and get emotionally drawn into their activities [45]. They also put unreasonable onus on themselves while put it away their own physical and emotional needs in a price to provide the best for the family member [46].

According to this finding, those having an average monthly income < 1000 ETB were nearly four times more likely to develop depression as compared to their counterparts. This finding was in agreement with other studies conducted in China [4] and Asia [9]. This might be due to the economic problems in most developing countries: that cancer disease has a poor prognosis, and the greatest concerns of the primary caregivers were insufficient health insurance coverage and the high cost of treatment. Furthermore, a primary caregiving role presents other challenges such as unexpected costs spent on drugs, feeding, and other related expenses at the hospital and at home. Moreover, family members and even relatives try to help the patient wholeheartedly because they feel an extreme commitment toward the patient. Therefore, they wish to have much more money to meet all their patient's needs. Some studies have reported that patients with increased care needs imposed great economic hardship on their families. About 10% of the household income was spent caring for the patient, resulting in the family taking out loans, getting second jobs, and finding other means to pay for healthcare costs.

According to this study was found to be that, primary caregivers of cancer patients with mixed therapy were nearly four times more likely to develop depression as compared to primary caregivers whose cancer patients with chemotherapy only. This finding was in line with other studies conducted in China [4]. This might be due to the cancer disease having a poor prognosis, and cancer treatments by themselves exposed patients to physical and psychological stress; so factors affecting the physical and psychosocial well-being of cancer patients influence family members too [31]. It also might be emotional distress, poor performance status of a patient, and Patient with mixed treatment may be indicated that patients had poor disease progresses [47]. Further, primary caregivers have varying emotional reactions to patient symptoms, which can cause distress as the patient’s health declines [48].

### Limitation

The current study focused on only primary caregivers who accompanied patients to cancer centers were assessed and this study did not show the level of depression. Most variables specifically behavioral related variables were self-reported and therefore may be affected by social desirability bias or defensive reactions.

## Conclusion

The prevalence of depression among primary caregivers of adult cancer patients was significant. Being female, having an average monthly income < 1000 ETB and primary caregivers of cancer patients with mixed therapy (Surgery + Hormonal therapy, Surgery + Chemotherapy, and Surgery + Chemotherapy + Hormonal therapy) were factors associated with caregiver depression. Its better, health care providers in oncology treatment facilities need to recognize and screen, give special attention to female caregivers, having burden of care and caregivers of a cancer patient with mixed therapy to reduce the risk of developing depression.

## Data Availability

All relevant data are available within the manuscript.

## Abbreviations

BDI: Back’s Depression Inventory
CEDS: Center of Epidemiological Depression Scale
ECOG: Eastern Cooperative Oncology Group performance
ETB: Ethiopian Birr
FHCSH: Felege Hiwot Comprehensive Specialized Hospital
GUCSH: University of Gondar Comprehensive Specialized Hospital
M.I.N.I: Mini-International Neuropsychiatric Interview
MDD: Major Depressive Disorder
OPD: Out-Patient Department
PHQ-9: Patient Health Questionnaire 9 Items
QoL: Quality of Life
SSA: Sub Saharan Africa
